# The impact of overweight and obesity on health-related quality of life in childhood – results from an intervention study

**DOI:** 10.1186/1471-2458-8-421

**Published:** 2008-12-23

**Authors:** Nora Wille, Michael Erhart, Christiane Petersen, Ulrike Ravens-Sieberer

**Affiliations:** 1 University Clinic Hamburg-Eppendorf, Research Group "Child Public Health", Center for Obstetrics and Pediatrics, Department of Psychosomatics in Children and Adolescents, Building W 29, Martinistr. 52, 20246 Hamburg, Germany; 2 Moby Dick Network, c/o Präventionszentrum Dr. Christiane Petersen, Lilienstraße 36, 20095 Hamburg, Germany

## Abstract

**Background:**

The negative impact of overweight (including obesity) and related treatment on children's and adolescents' health-related quality of life (HRQoL) has been shown in few specific samples thus far. We examined HRQoL and emotional well-being in overweight children from an outpatient treatment sample as well as changes of these parameters during treatment.

**Methods:**

In a cross-sectional design, self-reported HRQoL of 125 overweight (including obese) children who contacted a treatment facility, but had not yet receive treatment, were compared to 172 children from randomly selected schools using independent two-sample t-tests. Additionally, in a longitudinal design, the overweight children were retested by administering the same questionnaire at the end of the intervention (after one year). It included measures such as the body mass index (BMI), the general health item (GHI), the KINDL^R^, and the Child Dynamic Health Assessment Scale (ChildDynHA). Comparisons were based on dependent t-tests and the Wilcoxon signed-rank test.

**Results:**

Overweight children showed statistically significant impairment in the GHI (Cohen's d = 0.59) and emotional well-being (ChildDynha) (d = 0.33) compared to the school children. With respect to HRQoL, the friends dimension of the KINDL^R ^was significantly impaired in the overweight group (d = 0.33). However, no impairment was found for the total HRQoL score or other KINDL^R ^subdimensions. Regarding the longitudinal part of our study, most of the children improved their BMI, but the majority (87.5%) remained overweight. Nevertheless, the participants' perceived health, emotional well-being, and generic as well as disease-specific HRQoL improved during intervention.

**Conclusion:**

The findings emphasize the importance of patient-reported outcomes such as HRQoL. Even though overweight and obesity might accompany most of the children throughout their lifetime, the impairment associated with this chronic condition can be considerably reduced. Opportunities of health promotion in overweight/obese children and adolescents are discussed.

## Background

The increasing prevalence rates of childhood obesity are consensually recognized as a striking public health problem that threatens children's health considerably [1]. The phenomenon occurs especially, but not only, in the industrialized Western world and is often described as an epidemic [2,3]. In Germany, the prevalence rate of overweight is 15% in 3- to 17-year-olds, with 6.3% of that age group being obese [4]. There is overwhelming evidence that overweight and obesity are associated with an increased risk for a number of health conditions [5] as well as reduced life expectancy [6]. Even though the elevated disease burden and mortality mainly affect the adult population, serious somatic comorbidities increasingly appear during adolescence [7]. At the same time, treatment of obesity seldom proves effective in terms of a clinically relevant permanent weight loss [8].

The fact that obesity is predominantly a life-long condition emphasizes the importance of quality of life research in this field. Chronic health conditions require descriptions of health status and treatments beyond their impact on survival time. Thus, examining the condition's and/or its treatment's influence on the patient's life and well-being in terms of health-related quality of life (HRQoL) becomes important [9]. Knowledge of HRQoL can contribute to a better understanding of the patients' needs, an improvement in care, and a better evaluation of treatment. Furthermore, since the likelihood of attaining considerable long-term weight loss is small, emphasizing the physical, emotional, and social advantages of small weight reductions can support the patients' satisfaction with treatment outcomes [10].

Knowledge regarding the impact of overweight and obesity on HRQoL in pediatric patients is still limited [11]. One reason for this is that HRQoL research in children and adolescents has long been neglected [12]. Furthermore, the striking prevalence rates of childhood obesity are part of a trend that did not receive much attention until the past decade. However, HRQoL is of particular interest in overweight and obese youth since, at younger ages, psychosocial impairments are more prevalent than somatic comorbidities [13]. Therefore, reliable assessment of the global impairment associated with overweight and obesity is especially important in this age group.

Several studies of children and adolescents have shown a negative impact of overweight and obesity on HRQoL [14-17]. In contrast to overweight-associated impairments in adults, which mainly affect physical well-being [11], findings indicate a different type of impact in younger age groups. Beyond poorer ratings of subjective general health [18], limitations were primarily found in the psychosocial dimensions of HRQoL [14,15,19]. Considerable impairment was found in clinical as well as community-based study samples, especially regarding the social functioning domain [14,15].

Since research on overweight adults showed that HRQoL varies with treatment-seeking status and treatment intensity [20], more detailed knowledge regarding HRQoL in specific pediatric treatment samples is needed. There is especially a lack of knowledge regarding overweight and obese children enrolled in outpatient programs and the effects of this type of treatment. Outpatient programs play an important role in health care. They are not only connected to lower costs but also easier to integrate into the patients' lives, and, therefore, they are the most realistic treatment option for the majority of overweight and obese children.

In order to better understand HRQoL and well-being in young overweight outpatients, the present paper focuses on two questions regarding appropriate care and treatment [9]: (1) Which specific impairments are associated with overweight and obesity in this particular treatment group, and how are they subjectively represented? and (2) How does the treatment intervention influence the children's HRQoL?

In order to examine the first question in a German population, an overweight and obese outpatient sample of children and adolescents who had not yet begun treatment was compared to a sample of normal-weight schoolchildren within a cross-sectional comparison. Regarding the second question, in a longitudinal comparison, the overweight and obese outpatient sample was retested after the completion of the treatment program.

## Methods

### Sample description and procedure

#### Treatment sample

The group of overweight (including obese) children and adolescents included 125 respondents (61 female/64 male) from Hamburg aged 6 to 16 years, with a mean age of 11.02 years (SD = 2.41). Of this sample, 93% of the children were born in Germany; however, 39% reported that languages other than German were spoken at home. Sixty percent of the children rated the financial circumstances of their family as good, 33% as average, and 7% as poor. The children had signed up for a one-year outpatient treatment program between September 2003 and August 2004. Of the children, 97.5% were overweight in terms of exceeding the 90^th ^percentile, 79.9% of them were obese (exceeding the 97^th ^percentile), and 32.8% were extremely obese (their BMI was above the 99.5^th ^age- and gender-specific percentile) [21]. The 2.5% of the children who did not exceed the 90^th ^percentile were only slightly below it and were therefore admitted for treatment as well.

The treatment included participation in the one-year German outpatient program "Moby Dick". The program consists of attendance in a group that supports a balanced diet and regular exercise as well as the psychosocial well-being of the participants, and it aims to stabilize or reduce the participants' weight in the long-term. The children visited the group one afternoon per week for three hours. During these afternoons, the children first took part in an athletic program for 1.5 hours that aimed to create playful, success-oriented experiences by means of safe, physically active games with special attention to preventing injury. Afterwards, the children received lessons on nutrition, cooked together, and participated in role playing and other pedagogic interventions to enhance self-esteem and appropriate problem-coping behaviors. During all school holidays, a program was run between three and five days a week, with one compulsory date each week. The holiday program aims to promote physical activity during leisure time and to support new peer relationships through excursions (e.g. hikes, going swimming), workshops, games, and athletic competitions. The children register to participate in the different activities, which are conducted in part by external cooperating partners such as athletic clubs or youth clubs. In addition to the child-centered part of the program that included at least 52 weekly appointments during one year, the parents and further carers (e.g. grandparents, guardians) participated once a month in a meeting and received training in order to support the progress of their children. The sessions covered topics such as nutrition, medicine, physical activity, strengthening personality, and solving conflicts. Six meetings in the one-year program were parent-child afternoons.

The overweight children included in the cross-sectional comparison were contacted again one year after they completed the questionnaire for the first time. Of the 125 overweight children from the first data collection (T1), 80 (64%) participated in the second data collection. The longitudinal comparison refers to these 80 children who completed the questionnaire for a second time after treatment. The respondents included 42 girls and 38 boys 7 to 18 years old (m = 12.58; SD = 2.26). The participants of the second data collection (T2) had all completed the program, but no detailed information on the regularity of their attendance was available. The comparison of the 80 respondents of T2 with the 45 non-respondents of T2 showed that they did not differ in age, gender, weight status (T1), perceived health (T1), or disease-specific HRQoL (T1). However, the respondents of T2 scored significantly higher on the generic HRQoL (KINDL^R ^total score) at the beginning of the treatment than the non-respondents (d = 0.48) and, therefore, represent a positively biased selection.

#### School sample

The questionnaire was completed by a group of 193 children. Of these children, 21 (14.5%) were overweight (n = 16) or obese (n = 5) as determined by a BMI exceeding the 90^th ^or 97^th ^age- and gender-specific percentile of the German reference population [21], respectively. These children were excluded from the comparison. The 172 remaining children of the school sample were balanced in terms of the gender ratio (88 female/84 male). Their age ranged from 7 to 17 years, with a mean age of 11.88 (SD = 2.45). Of these children, 92% were born in Germany, but 38% reported that not only German was spoken at home. Fifty-eight percent of the children rated the financial circumstances of their family as good, 30% as average, and 12% as poor. They were pupils in grades 2 through 10 of five randomly selected schools in Berlin (three elementary schools and two high schools). In these schools, in all classes that were available for participation, an information letter was distributed to the parents. The children were temporarily excused from lessons, and they completed the questionnaires in a separate room.

The parents of all participants gave their informed consent to participate in the study. The study was approved by the ethics committee of the University of Hamburg, Germany. Descriptions of the samples are given in Table 1.

**Table 1:** Sample description: cross-sectional and longitudinal comparison

	**Cross-sectional**		**Longitudinal**	
	Overweight T1	School Sample	Overweight T1	Overweight T2
n	125	172	80	80
Age	6–16	7–17	6–16	7–18
	11.02 (SD = 2.41)	11.88 (SD = 2.45)	11.29 (SD = 2.29)	12.58 (SD = 2.26)
Sex	61 female	88 female	42 female	42 female
	64 male	84 male	38 male	38 male

#### Instruments/Measures

As a measure of overweight and obesity, the body mass index (BMI) was calculated from self-reported height and weight (kg/m^2^) and interpreted according to the gender- and age-specific cut-off points from German reference data [21]. According to current German guidelines, we used the 90^th ^and 97^th ^percentiles to identify overweight and obesity, respectively. Children who exceeded the 99.5^th ^percentile were classified as extremely obese. Additionally, standardized BMI z-scores were calculated [22] based on German reference data [21].

As a measure of perceived overall subjective health, the general health item (GHI) was included in the questionnaire ("In general, how would you say your health is?", with the answer options of "excellent", "very good", "good", "fair", and "poor"). The scores range from 1 to 5, with higher values indicating better subjective health.

To measure HRQoL, the KINDL^R ^Questionnaire [23] was administered. The generic KINDL^R ^Questionnaire was specifically designed and validated for use in children and adolescents, and it has proven to be highly reliable and sensitive to change [24]. It assesses the respondent's HRQoL by means of 24 Likert-scaled items in six dimensions: physical well-being, psychological well-being, self-esteem, family, friends, and everyday functioning (school or kindergarten). Additionally, the disease-specific obesity module of the KINDL^R ^Questionnaire was administered. It measures the respondent's quality of life regarding his or her obesity via a filter question and 12 further items. The scores on each KINDL^R ^subdimension and the total score were transformed to values between 0 and 100, with higher values indicating better HRQoL.

To assess the emotional well-being of the children, the Child Dynamic Health Assessment (ChildDynHA) Scale [25,26] was employed. It comprises nine Likert-scaled items that focus on the frequency of negative feelings such as sadness, unhappiness, concern, or anxiety as well as positive feelings such as happiness, zest for life, fun, and liking oneself. The scale was developed based on item response theory from a pool of items of widely used HRQoL instruments. The total score of the ChildDynHA Scale was transformed to values between 0 and 100, with higher values indicating greater emotional well-being.

### Statistical Analysis

To assess differences in subjective health between the treatment sample and the school sample, student's t-tests were conducted. Due to the skewed distributions, the results were controlled by conducting Man-Whitney-U-Tests, and in order to control for age and sex, an analysis of variance with age and sex as covariates was conducted to confirm the results. In order to detect a standardized mean difference of d = 0.5 with a statistical power of p = 0.8 and a two-tailed alpha of p = 0.05, a sample size of n = 64 for each group is required. In order to detect a smaller effect of d = 0.3, n = 176 is required. Since an insufficient number of overweight children presented for treatment, small effects can be detected with a power of only p = 0.72. However, the sample size in this study allows analyses for each gender with enough power to detect moderate effects. To test for changes in weight status during the intervention, a Wilcoxon signed-rank test was conducted. Paired samples t-tests were calculated to assess changes in BMI z-scores, perceived health, HRQoL, and emotional well-being. The actual sample size of n = 80 provides a power of p = 0.99 to detect a moderate effect. Since the sample size of n = 90 (which is necessary to detect a standardized mean difference of d = 0.3 with a statistical power of p = 0.8) could not be obtained, the sample provides a power of only p = 0.76 to detect small differences.

Pearson's correlation coefficient was calculated between the BMI z-score change and the improvement in HRQoL parameters. A moderate correlation of r = 0.3 can be detected with a power of p = 0.79 given the actual sample size. Descriptive statistics were calculated regarding changes in disease-specific HRQoL in children who managed to lose weight and those whose weight status remained unaffected or deteriorated.

P-values < 0.05 were considered statistically significant. Effect sizes (d) were given according to Cohen [27]. Effect sizes of 0.2 <d< 0.5 were classified as small, those from d = 0.51 to d = 0.8 as moderate, and d > 0.8 as large. For the calculation of effect sizes in the cross-sectional comparison, the mean difference was divided by the standard deviations of the school children. In the longitudinal analysis, the mean difference was divided by the standard deviations of the T1 overweight sample. Analyses were performed using the statistical software SPSS (Statistical Package for the Social Sciences; version 14). Power calculations were conducted using the software Power and Precision.

## Results

### Cross-sectional comparison

The results of the comparison between the overweight sample and the school sample are provided in Table 2. Compared to the school sample, the overweight treatment sample reported significantly poorer subjective health (Cohen's d = 0.59). Furthermore, the overweight sample obtained significantly lower scores on the ChildDynHA Scale as well as on the KINDL^R ^friends subdimension. Both differences can be interpreted as small effects (Cohen's d = 0.33), indicating impaired well-being of the overweight children.

**Table 2:** Differences between the treatment sample and the school sample

	**School sample**	**Overweight sample**	**T**	**df**	**p**	d**
General Health Item*	**3.73 **(0.97) n = 172	**3.16 **(0.94) n = 123	**5.06**	**293**	**.000**	**.59**
ChildDynHA*	**74.44 **(14.81) n = 161	**69.49 **(18.84) n = 122	**2.39**	**224**	**.02**	**.33**

**KINDL^R^**	(n = 165)	(n = 123)				
Physical Well-being	71.97 (19.05)	71.04 (17.37)	0.43	286	.67	
Psychological Well-being	80.11 (14.13)	78.15 (16.70)	1.05	237	.29	
Self-esteem	58.37 (20.40)	57.77 (20.87)	0.24	286	.81	
Family	80.49 (18.54)	80.84 (17.33)	0.16	286	.87	
Friends*	**76.93 **(16.09)	**71.60 **(21.28)	**2.33**	**219**	**.02**	**.33**
School	66.62 (20.81)	70.12 (19.64)	1.45	286	.15	

Total	72.42 (11.98)	71.59 (12.79)	0.56	286	.57	

* significant differences are printed in bold

** effect sizes are only given in case of significant difference

No statistically significant difference between the overweight children and the non-overweight children was observed in the KINDL^R ^total score. At the level of KINDL^R ^subdimensions, we also find, with the exception of poorer judgment in the friends dimension, small and statistically non-significant differences. All of these results were confirmed by a univariate analysis of variance controlled for age and sex, which found significant differences exclusively with respect to the general health item (F = 19.34; p < 0.001), the KINDL^R ^friends subdimension (F = 7.27; p < 0.007), and the ChildDynHA Scale (F = 8.57; p < 0.004).

To examine potential differences between the sexes, the same analysis was conducted separately for girls and boys. The comparison of the school girls with the overweight girls showed a significant impairment of the latter in self-reported subjective health (d = 0.49; p = 0.003) and emotional well-being (d = 0.38; p = 0.048). Furthermore, there was a statistical trend in the KINDL^R ^subdimension psychological well-being (d = 0.31; p = 0.094), whereas in the friends dimension, no trend was detected (d = 0.31; p = 0.129). The overweight boys showed significant impairment only in subjective health (d = 0.70; p < .001) and a statistical tendency in the KINDL^R ^subdimension friends (d = 0.35; p = 0.081). Their emotional well-being was not significantly impaired (d = 0.31; p = 0.121).

### Longitudinal comparison

**Figure 1:**
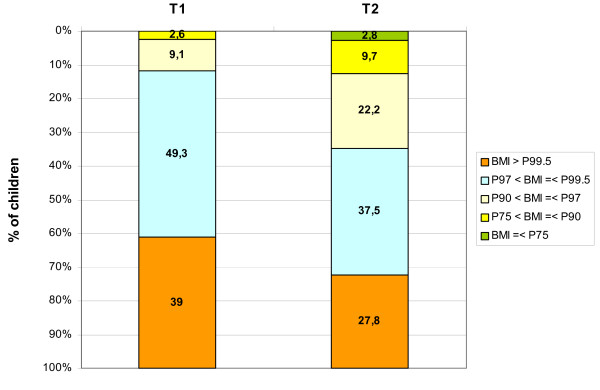
**Changes in age- and gender-specific BMI percentiles (P) from T1 to T2**. Referring to German age- and gender-specific reference data [21].

Concerning the weight/height relation expressed by the BMI, a significant decline between the first and the second data collection in the overweight and obese sample can be shown (figure 1). At T2, the percentage of non-overweight children (i.e., falling short of the 90^th ^age- and gender-specific percentile) rose from 2.6% to 12.5%. The percentage of obese children (exceeding the 97^th ^percentile) was reduced from 88.3% to 65.3%, and the percentage of extremely obese individuals decreased from 39% to 27.8%. This change in the BMI age- and gender-specific percentiles [21] was statistically significant (p < .001). Furthermore, a comparison of BMI z-scores shows a statistically significant decline from T1 to T2 (p < 0.001).

The changes in perceived health, emotional well-being, and HRQoL during treatment are presented in Table 3. A significant increase on the general health item from before to after participation in the outpatient program can be observed, indicating greater subjective health after treatment, with a moderate effect (d = 0.58). Furthermore, the ChildDynHA Scale of emotional well-being shows a significant increase that corresponds to a small effect (d = 0.39).

**Table 3:** Changes in psychosocial health-related parameters during intervention

	**N**	**Overweight T1**	**Overweight T2**	**T**	**df**	**p**	d**
General Health Item*	76	**3.25 **(0.88)	**3.76 **(0.95)	**3.61**	**75**	**.001**	**.58**
ChildDynHA*	78	**72.11 **(16.68)	**78.68 **(17.85)	**2.97**	**77**	**.004**	**.39**

**KINDL^R^**							
Physical Well-being	79	73.50 (15.51)	76.94 (18.20)	1.59	78	.12	
Psychological Well-being	79	80.06 (15.28)	83.78 (14.59)	1.87	78	.07	
Self-esteem	79	59.49 (18.40)	65.61 (23.44)	1.88	78	.06	
Family	78	82.77 (16.95)	82.55 (17.21)	0.10	77	.92	
Friends*	77	**74.11 **(19.47)	**79.46 **(16.78)	**2.10**	**76**	**.04**	**.27**
School	76	71.71 (19.77)	70.02 (19.33)	0.65	75	.52	
Total*	75	**73.36 **(12.08)	**76.80 **(13.24)	**2.12**	**74**	**.04**	**.28**
Obesity module*	68	**66.44 **(17.94)	**75.45 **(15.97)	**5.15**	**67**	**.000**	**.50**

* significant differences are printed in bold

** effect sizes are only given in case of significant difference

Concerning the changes in HRQoL during treatment, we found significant improvements in the KINDL^R ^total score. Regarding the KINDL^R ^subdimensions, the only statistically significant improvement was the increase of the KINDL^R ^friends score, while in other subdimensions, statistical tendencies were observed (physical well-being, psychological well-being, self-esteem). An especially pronounced increase was observed in the KINDL^R ^obesity module. The effect sizes concerning the improved scores were small in the friends scale (d = 0.27) and in the total score (d = 0.28), while in the KINDL^R ^obesity module (d = 0.50) and the general health item, a moderate effect (d = 0.58) was found.

The observed improvement of the generic and disease-specific HRQoL scores is not significantly associated with the improvement in BMI. Pearson's correlation coefficients between the changes in BMI z-score and changes in the improved parameters do not reach statistical significance (KINDL^R ^total score: r = 0.054, p = 0.653; KINDL^R ^obesity module: r = -0.145, p = .272; KINDL^R ^friends scale: r = -0.008, p = 0.948; GHI: r = -0.203, p = 0.105; ChildDynHA: r = -0.090, p = 0.471). The data even show that all children, even those with worsened weight status, benefit from the intervention in terms of increasing disease-specific HRQoL. However, even though the KINDL^R ^obesity module shows the highest increase in children who improved their weight status (mean increase +11.47) compared to those who maintained their weight status (mean increase +8.95) and to those whose weight status deteriorated (mean increase +7.50), the differences in these increases are not statistically significant.

## Discussion

The comparison of the school sample with the treatment-seeking overweight children showed impairment of the latter regarding their perceived health, social well-being, and emotional well-being. However, further dimensions of HRQoL such as physical well-being, psychological well-being, self-esteem, family and school life, as well as overall HRQoL, were not significantly impaired.

This pattern of impairment and non-impairment in the outpatient treatment sample corresponds to previous studies of clinical and community samples that also reported a specific impact of overweight/obesity on the social domain [14,15]. The reported disadvantages in social life must be taken seriously. Previous research has found that lower self-acceptance in obese adults was fully mediated by the experience that one has been discriminated against due to weight status [28]. Thus, the social impairment might be interpreted as a starting point leading to negative self-evaluations in the future.

Interestingly, regarding the impact of overweight on emotional well-being, a significant impairment is revealed by the ChildDynHA Scale, whereas the KINDL^R ^psychological well-being dimension is not affected. Obviously, the differentiated assessment of positive and negative emotions in the former is sensitive to the emotional trouble of overweight and obese children, whereas the KINDL^R ^psychological well-being scale, with only four items (ChildDynHa: 9 items), cannot capture these problems. The different results on the two scales demonstrate the importance of the choice of instruments when dealing with psychological, emotional, or psychosocial aspects of childhood obesity. Especially in mildly impaired samples, reduced well-being can be missed if instruments primarily focus on problems or negative moods and do not sufficiently address positive emotions.

The significant impairment of perceived subjective health clearly shows that the overweight and obese children realize that their weight negatively impacts their general health, which confirms the results of previous studies [18]. Nevertheless, the fact that we did not find significant differences in the KINDL^R ^physical dimension supports the suggestion that, at earlier ages, the somatic consequences are still less important compared to psychosocial consequences, while physical domains of functioning are primarily impaired in adults [11].

Overweight-associated impairment was similar in girls and boys for almost all dimensions. However, regarding emotional and psychological well-being, evidence indicating a higher emotional burden in girls was found. Nevertheless, this result must be handled with care since only moderate effects are likely to be detected in the sex-specific analyses. Therefore, lower power likely accounts for the lack of significant differences in boys.

In general, the number and the extent of overweight-associated impairment in the sample under study are smaller than those previously found in inpatient populations [e.g., [14]]. The milder impairment in this outpatient sample is in line with the idea that HRQoL varies with treatment intensity [20]. Similarly, the higher psychosocial impairment in our treatment-seeking sample as compared to previous population samples [18] is in accordance with this assumption. However, there are also findings from community-based samples that indicate a more comprehensive overweight-associated impairment than does the current study [15]. It does not suffice to refer to the questionable comparability between different countries and measures in order to interpret the observed variability of results. Instead, crucial aspects such as the access to treatment and its demands on the patients must be considered when discussing varying HRQoL in overweight and obese subgroups. For example, the connection between subjective impairment and treatment status might be weaker in children since participation in a treatment program is highly dependent on the parents' perception of the problem.

Furthermore, children who join a program that requires permanent participation of children and parents may be those overweight children with stronger familiar resources. Their participation presupposes a family that realizes the problematic nature of weight status and that is motivated to integrate the program into family life. Therefore, overweight children enrolled in comparable outpatient programs might be more likely to be a positive biased selection than a negative biased selection compared to the general population of overweight children. Further research is needed to determine the extent to which not only risks but also protective factors vary systematically in different pediatric overweight and obese samples.

The improvement of subjective health, emotional well-being, and generic as well as disease-specific HRQoL during treatment is particularly interesting since the change in BMI, although it decreased significantly, was small. The statistical tendencies of physical and psychological well-being as well as self-esteem hint at possible further pre-post improvements that might have been undetected due to limited power. Less than 13% of the children reached a normal weight (i.e., fell short of the 90^th ^age- and gender-specific percentile). After treatment, 65.3% of them remained obese, and 27.8% remained extremely obese. Unfortunately, this exemplifies the difficulties of weight loss that the majority of overweight and obese persons face. Regarding this problem, a remarkable improvement in HRQoL can be a compensation for not fully achieving weight loss [10], and it can be helpful for maintaining the motivation to continue lifestyle modifications. Likewise, the psychosocial improvements indicate greater opportunities for the children in terms of healthy development, and such improvements counteract their higher risk of mental health problems [29]. In particular, the improvement in the KINDL^R ^obesity module shows that the specific impairments connected to obesity can be strongly reduced.

We are aware of the fact that the lack of an association between weight loss and increasing HRQoL might be attributable to the limited power of this analysis. In order to detect small correlations, a larger sample size would have been necessary. However, since in our sample a moderate (r = 0.3) or strong (r = 0.5) correlation would have been detected with an adequate power of p = 0.79 and p = 0.99, respectively, it is unlikely that such a sizeable association exists. Similarly, the comparison of groups with different weight status development can only be of a descriptive nature. Nevertheless, the observation of increased HRQoL despite limited success in weight loss contains a positive message. Although some of the children will not be able to reduce their weight significantly, the suffering associated with their condition can be reduced. Regarding the limited success of obesity treatment in terms of weight loss, it would be reasonable to identify interventions that promote HRQoL and emotional well-being in participants who do not manage to alter their weight status. Since lower levels of depressed mood or anxiety and higher self-esteem positively impact healthy lifestyle choices [30], the implementation of health promotion strategies is indispensable in interventions that focus on weight loss in order to reduce the health consequences of childhood obesity.

However, the limitations of the study should be considered. Due to the self-reporting of height and weight, upon which we relied without collecting actual measurement, the reliability of the BMI data is questionable [31]. Consequently, the absence of correlations between weight loss and psychosocial improvements may be a result of biased BMI data. Furthermore, since the self-reported BMI is subjected to a systematic underestimation [31], the school children who were considered to be normal weight might include some overweight or even obese children.

Other limitations of the study are connected to the lack of a randomly controlled study design. In the longitudinal part of our analysis, we cannot attribute the improvements of the overweight children to the intervention program. Even if our comparison between pre- and post-treatment status allows the conclusion that the participants improved after intervention, a causal relationship cannot be stated. This is exacerbated by the fact that the children who filled in the questionnaire at the second timepoint were at an advantage when the intervention started, and, therefore, a selection bias influencing the results must be assumed. Furthermore, we were not able to ensure that all of the overweight children who filled in the questionnaire at the second timepoint after one year had participated in the program regularly. However, the groups are characterized by a high commitment. If a child could not attend a group meeting, the parent was required to excuse the child beforehand. Since parents only receive reimbursement for the program fees from their health insurance in the case of regular attendance (80% of appointments), there is also a financial incentive to attend the group regularly. In general, due to the small sample sizes, some results of this study are of limited explanatory power.

## Conclusion

This study showed a significant impairment of perceived health as well as social and emotional well-being in German overweight and obese children participating in an outpatient program. The relatively mild impairment of the examined outpatient sample supports previous research indicating that the impact of obesity on HRQoL varies in different obese samples, although this finding has limited transferability to a pediatric population. The results of this study correspond to previous research indicating primarily psychosocial consequences of childhood obesity.

Although no causal relationship can be stated, the results of our study suggest a possible benefit of participating in the program in terms of subjective health, emotional well-being, and generic as well as disease-specific HRQoL. This improvement was observed even though the majority of the sample remained overweight. Improvements of HRQoL and well-being during intervention despite the absence of weight loss point to an appropriate way to address overweight and obesity in patients who will deal with this chronic condition throughout their lives. More information on programs that enable children to lead full lives regardless of weight is needed.

## Abbreviations

HRQoL: Health-related quality of life; BMI: Body Mass Index; GHI: General Health Item; ChildDynHA: Child Dynamic Health Assessment Scale.

## Competing interests

The authors declare that they have no competing interests.

## Authors' contributions

NW was extensively involved in the acquisition and interpretation of data, performed statistical analysis and interpretation, and drafted the manuscript. ME made substantive contributions to statistical analysis and interpretation of data and critically revised the manuscript for important intellectual content. CP made substantial contributions to the conception and design of the study, organized data collection, and critically reviewed and edited the manuscript. URS made substantive contributions to the conception and design of the study, analyzed and interpreted data, and critically revised the manuscript for important intellectual content. All authors read and approved the final manuscript.

## Pre-publication history

The pre-publication history for this paper can be accessed here:


